# Parents’ Reports of Preschoolers’ Diets: Relative Validity of a Food Frequency Questionnaire and Dietary Patterns

**DOI:** 10.3390/nu11010159

**Published:** 2019-01-13

**Authors:** Liisa Korkalo, Henna Vepsäläinen, Carola Ray, Essi Skaffari, Reetta Lehto, Helena Henrietta Hauta-alus, Kaija Nissinen, Jelena Meinilä, Eva Roos, Maijaliisa Erkkola

**Affiliations:** 1 Department of Food and Nutrition, University of Helsinki, P.O. Box 66, 00014 Helsinki, Finland; liisa.korkalo@helsinki.fi (L.K.); essi.skaffari@helsinki.fi (E.S.); maijaliisa.erkkola@helsinki.fi (M.E.); 2 Folkhälsan Research Center, Topeliuksenkatu 20, 00250 Helsinki, Finland; carola.ray@folkhalsan.fi (C.R.); reetta.lehto@folkhalsan.fi (R.L.); jelena.meinila@helsinki.fi (J.M.); eva.roos@folkhalsan.fi (E.R.); 3 Children’s Hospital, Pediatric Research Center, University of Helsinki and Helsinki University Hospital, Biomedicum 2 C, P.O. Box 705, 00020 HUS Helsinki, Finland; helena.hauta-alus@helsinki.fi; 4 Seinäjoki University of Applied Sciences, Kampusranta 11, 60101 Seinäjoki, Finland; kaija.nissinen@seamk.fi; 5 Department of Obstetrics and Gynecology, University of Helsinki and Helsinki University Hospital, P.O. Box 22, 00014 Helsinki, Finland; 6 Department of Public Health, University of Helsinki, P.O. Box 20, 00014 Helsinki, Finland

**Keywords:** validation study, dietary assessment methods, food diary, cross-classification, children, whole diet, preschool, DAGIS Study

## Abstract

The accurate assessment of food consumption is crucial in nutritional studies. Since modern nutrition science has become more interested in diet as a whole, studies validating food frequency questionnaires (FFQs) and exploratory dietary patterns are needed. We aimed at examining the relative validity of a 47-item FFQ against three-day food records among three- to six-year-old Finnish children, as well as investigating the consistency of the dietary patterns derived using the principal component analysis (PCA), with food record and FFQ data as inputs. We conducted the PCA without forcing the food record data to match the FFQ items. Altogether, 75% or more of the participants were classified into the same or adjacent quarter of vegetables and fruits as well as sugary food consumption. Furthermore, the intake of folate and vitamin C increased linearly in the quarters of vegetable and fruit consumption, as did the intake of sucrose in quarters of sugary food consumption. Three fairly similar dietary patterns were identified from food records and FFQ data. Concerning the patterns, more than 70% of the participants were classified into the same or adjacent quarter. However, the Spearman correlation coefficients between the respective pattern scores were low (0.25–0.33). The FFQ showed acceptable validity when ranking food group consumption compared to food records. Additionally, the FFQ-derived dietary patterns were consistent with those derived using food record data.

## 1. Introduction

As food consumption cannot be measured objectively, researchers must rely on self-reported methods, such as food records, dietary recalls, or food frequency questionnaires (FFQs). FFQs are often used in large, epidemiological studies because of their cost-effectiveness and relatively low respondent burden [1,2]. Children under the age of 7 years are not able to report their own food consumption, and thus, the acquisition of dietary information is dependent on parents, other guardians, or early childhood educators, for instance [3]. Therefore, the accuracy of dietary assessment in children is dependent on the adults’ ability to reliably and validly report the children’s food consumption. The relative validity of an FFQ can be investigated by comparing the food consumption data obtained using an FFQ with the data obtained using a separate dietary assessment method. Food records are a good method for estimating the relative validity of an FFQ, because the two methods do not share the main sources of errors [4], but other reference methods, such as 24-hour recalls or plasma biomarkers, have also been used in studies validating FFQs among children [5].

Traditionally, validation studies have compared the consumption frequency of certain foods or food groups (e.g., fruits and vegetables, or beverages) or calculated nutrient intakes (e.g., calcium or energy intake) derived using two separate methods (see, for example, [6,7,8,9,10]). However, data-driven dietary patterns that are indicative of a person’s whole diet have become popular in nutritional epidemiology [11]. One of the most frequently used methods to derive dietary patterns is principal component analysis (PCA), which is based on intercorrelations between food items [12,13]. The extraction of dietary patterns using PCA requires, however, multiple subjective decisions, such as the selection of dietary variables, to be included in the analysis [14]. Furthermore, the dietary assessment method used to measure food consumption may affect the extracted patterns. Thus, the validation of PCA-derived dietary patterns is beneficial.

Data-driven dietary patterns are thought to reflect the usual food consumption of the participants. Thus, FFQs, which also describe food consumption in general, have mostly been used as inputs in the dietary pattern analysis. Since the late 1990s, several studies have compared FFQ-based dietary patterns with patterns derived from food record data among adults [15,16,17,18,19,20,21]. Only two studies have compared FFQ- and food-record-based patterns among children [22,23]. However, the studies have matched each food in the food records with an FFQ item, possibly resulting in artificially increased correlation coefficients [20].

The objective of the current study was two-fold, namely: (1) to study the relative validity of a non-quantitative 47-item FFQ against a three-day food record (reference method) of preschool children by evaluating the ability of the FFQ to rank individuals in the same order according to food consumption as compared to the reference method, and (2) to study the consistency of the data-driven dietary patterns derived by PCA using two sources (food record and FFQ) of data. Our study broadly looked at the validity by examining both the direct (comparison of food consumption to food consumption) and indirect (comparison of food consumption to nutrient intake) validity of the FFQ, as well as the relative validity of the dietary patterns. However, we only measured food consumption outside of preschool.

## 2. Materials and Methods 

### 2.1. Study Participants

Increased Health and Wellbeing in Preschools (DAGIS) is a research project that aims to diminish socio-economic differences in preschool children’s energy balance-related behaviors in Finland (www.dagis.fi). As a part of the larger project, a cross-sectional study was conducted between September 2015 and April 2016, and the details of the sampling process are described in open access format [24]. In short, the cross-sectional study was conducted in eight municipalities. Altogether, 86 communal preschools (56% of those invited) consented to participate. These preschools operated from Monday to Friday and served three meals per day—breakfast, lunch, and an afternoon snack. Preschools operating 24 hours per day were not included in the sample. From the consenting preschools, all of the children in the target age of three to six years (*n* = 3592) and their families were invited to participate through an invitation letter. Children in preschools with a low participation rate (≤30% in each of the preschool groups for three to six year-olds) were excluded. The final sample consisted of 864 children (24% of those invited) from 66 preschools. A parent or legal guardian of each participating child provided written informed consent. The University of Helsinki Ethical review board in humanities and social and behavioral sciences reviewed the study on 24 February 2015, and found the study ethically acceptable (Statement 6/2015).

### 2.2. FFQ

We designed a short FFQ for measuring children’s food consumption during the previous week outside of preschool hours. The FFQ was developed to assess the children’s dietary quality in general, and specific attention was paid to capturing the consumption patterns of vegetables and fruits as well as sugary foods and beverages. The FFQ included 47 food items divided under seven headings, as follows: vegetables, fruits, and berries; dairy products; fish; meat and eggs; cereal products; drinks; and others (i.e., sweets and snacks). The questionnaire was available in both official languages of Finland (Finnish and Swedish) and in English; the English version of the FFQ is available online [25]. A shortened, 25-item version of the FFQ has been tested for reproducibility with mostly moderate or good intraclass correlation coefficients [26].

The participating families received a letter including the FFQ, a three-day food record, and detailed instructions by post. The instructions were to fill in the FFQ first and the three-day food record later, on pre-set dates. The time between the two methods was roughly one week. In the FFQ, the respondent (parent or legal guardian) reported how many times during the past week the child had consumed different foods outside preschool. We intentionally restricted the FFQ to not cover foods and drinks consumed during the preschool hours because the parents would not have been able to reliably report these foods. The time spent at preschool may vary from child to child as well as from day to day, and therefore, we did not specify which meals should be included or excluded in the FFQ. The FFQ included the following three answer columns: “not at all”, ‘times per week”, and “times per day”. The instruction was to either tick the “not at all” box or to write a number in one of the other columns. If two or more FFQ items were missing, nutritionists contacted the families and the missing items were completed if possible.

### 2.3. Three-Day Food Record Data

The study group assigned the exact dates (two weekdays and one weekend day) for each family to fill in the food record. These three days were not always consecutive days. In some cases, when the pre-set dates were unsuitable for the family, the parents contacted the study group and the dates were renegotiated. The instruction was to record all of the foods and beverages consumed by the child during the three days, except for consumption at preschool. We provided the families with a children’s food picture book, specifically designed for use in this project, to assist in portion size estimation [27,28]. The parents estimated portion sizes by using the food picture book or household measures, by weighing, or from package labels. The instruction was to list all of the ingredients of the composite dishes. For prepacked products, the exact brand and product name was to be given. Preschool personnel filled in a separate food record during preschool hours, but these data were not used in the analyses of this paper.

The larger DAGIS project had a specific focus on vegetables and fruits as well as sugary food consumption [29]; therefore, these food groups were given special attention in the food record checking process. Nutritionists checked all of the food records. If there were shortcomings in the information concerning vegetables, fruits, and berries, or sugar-containing foods or beverages, the nutritionists made follow-up phone calls or emailed the parents to complete missing details of these foods. As an example, if the parent had forgotten to record the type of yoghurt product, we asked if it was a natural yoghurt or sugar-sweetened yoghurt. Nutritionists entered the food record data using AivoDiet dietary software (version 2.2.0.0, Aivo Oy, Turku, Finland). AivoDiet software included the Fineli Food Composition Database Release 16 (2013). New food items were added into the database when necessary. The database includes recipes for typical Finnish mixed dishes. For each individual meal, the nutritionists either used a suitable recipe found in the database or created a new recipe according to parents’ reports. During data entry, each meal was tagged with a code indicating the place of consumption, which enabled us to remove the foods eaten at preschool from the analyses.

To compare the food consumption frequencies during the three-day food record period with the food consumption frequencies reported in the FFQ, we listed every food code used in the food record data and assigned them to the corresponding FFQ row(s). The food record data used in the analyses of this paper included a total of 2421 individual food codes (food items or mixed dishes). Each mixed dish food code was assigned to no more than three rows, according to the main ingredients. For example, a meat soup, which included carrots and potatoes, was assigned to the rows “red meat”, “potatoes”, and “cooked and canned vegetables”. Out of the 2421 food codes, 1714 codes were assigned to one FFQ row, 228 codes to two FFQ rows, and 96 codes to three FFQ rows. The rest (i.e., 383 codes) represented foods not covered by the FFQ, and these were not assigned to any FFQ rows. These codes included, for example, spices, savory pastries, and fat spreads.

### 2.4. Comparison of the FFQs and Food Records

For the comparison of the FFQs and food records, we used data from all of the children with their FFQs filled in with no more than six missing rows out of the 47 rows, and three days of complete data in the food record (*n* = 756). The number of families who filled in the FFQs for these children was 674; 594 families had one child in the sample, 78 families had two children in the sample, and two families had three children in the sample. The majority (*n* = 747; 99%) of these children were in the target age group of three to six years old, but seven children were two years old and two were seven years old. The differences in the age and sex distribution between the children included in the analysis and the children excluded from the analysis because of missing food consumption information (*n* = 108) were tested using the Student’s *t*-test and Chi-Squared test.

To test how well the FFQ was able to rank individuals in the same categories of food consumption frequency when compared to the reference method (food record), each child was assigned to one of four categories created according to the quartiles of the distribution. Note that we only considered food consumption outside of preschool hours. The categories were cross-tabulated, and the percentages in the same, same or adjacent, and opposite quarters were calculated. We also calculated the proportions of non-consumers and the weekly and daily consumers according to the FFQ. For foods with a high proportion of non-consumers, the majority of the participants were automatically assigned to the first category and the data were assigned into only two or three categories in total. This was true for both methods. Thus, some cells in the cross-tabulations remained empty.

To evaluate whether the food consumption frequency, as measured by the FFQ, was associated with the intake of key nutrients, as calculated from the three-day food records that indicate the consumption of specific types of foods, the mean intakes of selected nutrients were computed according to the consumption categories of selected food groups from the FFQs. We selected the food groups that the DAGIS project has a specific focus on (i.e., vegetables and fruit, and sugary foods and beverages). The consumption frequency was evaluated against the folate and vitamin C (vegetable and fruit consumption) and sucrose intake (sugary foods and beverages). In addition, we evaluated milk, which is typically consumed daily by Finnish preschoolers, against calcium intake. The food group frequency categories were created by summing the responses of the selected FFQ rows. Missing values were treated as zeros when summing the responses. We then categorized the food group consumption frequencies into quarters. Trend analysis was performed using linear regression. The food consumption frequency quarter was treated as a continuous predictor variable, and the nutrient intakes were square root transformed.

### 2.5. Comparison of Dietary Patterns

In order to identify the existing dietary patterns in the sample, we conducted separate PCAs using (1) the FFQ food groups and (2) food record data (all of the items consumed outside preschool) as inputs. All of the children with complete FFQ data (*n* = 758; 88% of all of the DAGIS participants) were included in the FFQ-based PCA, in which all of the 47 FFQ food items were used as input variables (see [30] for more details). For the food-record-based analysis, the food record codes were collapsed into 64 food groups based on the nutritional similarity of the foods. All of the children with food record data from three days and with no more than six missing FFQ rows (*n* = 756) were included in the analysis. Of these food groups, we excluded 17 because of low consumption (mean consumption less than 10 g in three days). Due to having no nutritional value and inconsistencies in reporting, we further excluded water from the analysis. Similarly, a mixed group containing foods that could not easily be classified into any of the existing food groups (e.g., spices, spice sauces, meal replacement products, and other miscellaneous foods) was excluded. Thus, the consumption of 45 food groups (g/day) were used as inputs in the food-record-based PCA (see Supplementary Table S1 for included and excluded food groups). IBM SPSS Statistics versions 22 (IBM Corp., Armonk, NY, USA) and 25 (IBM Corp., Armonk, NY, USA) were used in the PCA.

Based on the eigenvalues (minimum value of at least 1.5), scree plots, and interpretability of the components, we chose three components for the FFQ-based analysis and five components for the food-record-based analysis. The analyses were then rerun with forced three- or five-component solutions, respectively. The components were rotated with an orthogonal Varimax transformation, and standardized component scores for all of the components were calculated for each participant. Thus, the obtained component scores represented how closely the food consumption of each participant mirrored each of the empirically derived dietary patterns. The children with complete data for both methods (*n* = 705; 93% of those included in the comparison of FFQs and food records) were included in the dietary pattern analysis comparison. We used Spearman correlation coefficients to compare the FFQ- and food-record-based dietary pattern scores. In addition, we categorized the FFQ- and food-record-based dietary pattern scores into quarters and calculated the proportion of participants classified into the same quarter, same or adjacent quarter, or grossly misclassified into opposite quarters.

## 3. Results

The majority of the children (*n* = 742; 98%) had food record data for two weekdays and one weekend day, as planned. The rest (*n* = 14; 2%) had food record data for one weekday and two weekend days. A preschool day was defined as a day when the child ate at least one meal at preschool, and most children had food record data for two preschool days (*n* = 621; 82%). Others had either one (*n* = 108; 14%) or no preschool days (*n* = 27; 4%) included in the three-day food record. The children included in the current analysis did not differ from the excluded children in terms of age (Student’s *t*-test *p* = 0.89) or gender (Chi-Squared test *p* = 0.51).

### 3.1. Comparison of FFQs and Food Records

Table 1 shows the proportions of participants classified into same, same or adjacent, and opposite quarters, using the two dietary assessment methods. Depending on the FFQ food item, 60%–96% of the participants were classified into the same or adjacent quarter. The proportion of participants classified into the same or adjacent quarter were 72%–80% for vegetables and fruits (fresh fruit; fresh vegetables; cooked and canned vegetables; peas, beans, lentils, and soya). The respective proportions for sugary treats (ice cream; chocolate; sweets; cakes, cupcakes, sweet rolls, Danish pastries, pies, and other sweet pastries; sweet biscuits and cereals bars) were 70–81%, whereas the consumption of sugary everyday foods (flavored yoghurt; puddings; sugar-sweetened cereals and muesli; berry, fruit, and chocolate porridge with added sugar; berry and fruit soups with added sugar) were classified into the same or adjacent quarter in 71%–87% of participants. Regarding the consumption of sugary beverages (soft drinks; flavored and sweetened milk- and plant-based drinks; sugar-sweetened juice drinks), 69%–78% of the participants were classified into the same or adjacent quarter. The proportion of participants grossly misclassified was 10% or less for vegetables and fruits as well as for sugary foods. Of the individual food items, flavored nuts, almonds, and seeds; whole milk and sour milk; berry, fruit, and chocolate porridge; and reduced-sugar soft juices and soft drinks were most similarly reported, with 85% or more of the participants classified into the same quarter. Canned and frozen fruits, eggs, reduced-sugar soft juices and soft drinks, and dried fruit and berries were the least accurately classified, with 15%–22% of the participants grossly misclassified into opposite quarters.

The calculated mean daily vitamin C and folate intakes increased linearly in the quarters of vegetable and fruit consumption (Table 2). Similarly, calcium intake was positively associated with milk consumption frequency, whereas sucrose intake increased in the quarters of sugary treats, sugary everyday foods, and sugary beverage consumption.

### 3.2. Comparison of Dietary Patterns

The five food-record-based components explained altogether 19.7% of the variance in the sample (Supplementary Table S2), whereas the corresponding percentage in the FFQ-based analysis was 16.7% (for more details regarding the FFQ-based dietary patterns, please see [30]). Supplementary Table S2 also shows the loadings of foods into the components, percentage of variance explained by each component, and the eigenvalues in the food-record-based PCA. The food-record-based patterns were labelled “health-conscious”, “sandwich”, “sweets-and-treats”, “milk, potatoes, and minced meat”, and “pasta, minced meat, and fruit”, based on the foods loading positively to each component. The FFQ-based patterns were named “sweets-and-treats”, “health-conscious”, and “vegetables-and-processed meats”, and have been reported in more detail elsewhere [30]. The three strongest patterns identified using the two methods were very similar to each other, and there were relatively low, positive correlations between the pattern scores (Table 3). Regarding each of the dietary pattern scores, over 70% of the participants were classified into the same or adjacent quarter, whereas the proportion of participants classified into opposite quarters was 6%–9% for the three patterns (Table 4).

## 4. Discussion

The aim of the current study was to compare the food consumption frequencies and empirical dietary patterns derived with the following two methods: food records and FFQs. Regarding consumption frequencies, the percentage of participants classified into the same or adjacent quarter as measured with FFQs and food-records was 60%–96%. Considering vegetable and fruit consumption, 72%–80% of the participants were classified into the same or adjacent quarter in the current study, whereas the corresponding percentages for sugar-containing foods were 69%–87%. Furthermore, the calculated nutrient intakes for key nutrients increased linearly in the quarters of vegetable and fruit, and sugar-containing food consumption. In addition, we found dietary patterns of the same kind using FFQ and food-record food groups as input variables. To our knowledge, this is the first study to perform PCA separately based on FFQs and food records without forcing the food record food codes to match the FFQ rows, which could lead to overestimated correlation coefficients [20].

### 4.1. Relative Validity of the DAGIS FFQ

The percentages of participants classified into the same or adjacent quarter were the greatest for skimmed milk and sour milk (96%); 1% fat milk, semi-skimmed milk, and sour milk (94%); flavored nuts, almonds, and seeds (91%); and whole milk and sour milk (90%). In general, the food items that were used only by a small percentage of participants (less than 20% according to the FFQ) had the largest proportions of participants classified into the same or adjacent quarter. Among food items that were used by more than 20% of the participants at least once according to the FFQs, commercial baby foods and smoothies with no added sugar; plain almonds, nuts, and seeds; and soft drinks with added sugar were most similarly reported, as 77%–80% of the participants were classified into the same or adjacent quarter as measured with the FFQs and food records. By contrast, the relative validity of our FFQ to assess the consumption frequency of canned and frozen fruit, eggs, reduced sugar juices and soft drinks, and dried fruit and berries could partly be criticized, because 15%–22% of the participants were grossly misclassified into opposite quarters by the two methods. The difference in time spans (three days vs. seven days) might at least partly explain these misclassifications. In the future, it would be advisable to rephrase these items or to incorporate the foods into FFQ rows with a lower proportion of misclassification (for example, canned and frozen fruit could be incorporated into fresh fruit).

Our first aim was to compare the consumption frequencies reported using food records and FFQs. Considering vegetable and fruit consumption, 35%–68% of the participants were classified into the same quarter in the current study, whereas previous studies reported respective percentages ranging from 35%–49% for vegetable and fruit FFQ items [6,8,9]. The fact that the calculated intakes of vitamin C and folate increased linearly through the quarters of vegetable and fruit consumption frequency (Table 2) gives additional support to our finding that, compared to food records, the FFQ is able to rank the children acceptably according to food group consumption. Using a somewhat similar approach, Flood et al. reported an increasing trend in the calculated vitamin C intake for vegetable consumption, but not for fruit consumption [7].

Regarding sugary foods, the percentages of participants classified into the same quarter varied depending on the type of foods. The lowest percentages were observed for sugary treats (ice cream 37%; sweet biscuits and cereal bars 35%; sweet pastries 27%; sweets 20%; chocolate 33%). These are typically foods that are not consumed daily, and thus, their habitual consumption frequency may have been more accurately captured by the FFQ. On the other hand, as keeping a food record is known to affect food behavior [1], it may be that the parents withheld sugary treats during the three days the food records were kept. Previous studies have reported corresponding percentages ranging from 32%–37% [6,8,9]. However, the wide variation in food groupings between the studies limits the comparison. In the current study, 49%–87% of the participants were classified into the same quarter with regard to sugary everyday foods (berry and fruit soups; flavored yoghurt and quark; puddings; sugar-sweetened cereals and muesli; berry, fruit, and chocolate porridge), whereas the corresponding range for sugary beverages (flavored and sweetened milk- and plant-based drinks; sugar-sweetened juice drinks; soft drinks) was 37%–72%. The calculated sucrose intake increased linearly in the quarters of sugary treats, sugary everyday foods, and sugary beverages, suggesting a good indirect validity.

### 4.2. Relative Validity of the Dietary Patterns

Currently, explorative or data-driven dietary patterns are used frequently in modern nutrition science [11]. The dietary pattern approach has many advantages. First, the patterns provide us with a broader conception of food consumption and can add to our understanding of dietary behavior [31]. Second, the pattern approach is free of a priori assumptions concerning, for example, healthful diets, and describes the actual parallel presence of different patterns in the diets of the participants [12]. PCA is one of the most frequently used methods for dietary pattern analysis, and usually FFQ data are used as inputs for the analysis. The validity of the FFQs used to derive dietary patterns is often under study, but only a few studies have investigated the relative validity of the resulting dietary patterns among children [22,23].

To our knowledge, this is the first study to compare dietary patterns derived using two dietary assessment methods, without forcing the input variables to be similar (i.e., matching the foods from the food records into FFQ rows). The matching of the food items has resulted in the exclusion of a large number of foods that cannot be matched to each of the FFQ rows. For example, in a sample of middle-aged and elderly Swedish women, as much as 54% of the foods in the food records were excluded from the analyses [18]. Using this approach, the validity of the patterns is likely to be better than the actual validity [20].

In the present study, we found three quite similar dietary patterns using two dietary assessment methods (FFQs and food records). The pattern scores were correlated as statistically significantly, but the correlation coefficients were relatively low (0.25–0.33) [32]. Previous studies have reported correlations ranging from 0.32–0.74 among adults [15,16,17,18,19,20], and the correlations have been of the same order among adolescents [22] and toddlers [23]. Our lower correlation coefficients are probably due to not matching the foods from the food records to the FFQ rows. Instead, we conducted the PCA separately, using FFQ rows and food record data as inputs. Thus, we believe that the correlation coefficients in this study reflect the validity of the dietary patterns more realistically. However, more than 70% of the participants were classified into the same or adjacent quarter, according to the dietary pattern scores. Hence, it seems that despite the low correlation coefficients, the two dietary assessment methods can provide fairly similar dietary patterns and rank the participants similarly based on the dietary pattern scores.

### 4.3. Strengths and Limitations

The present study had several strengths. First, we had a large sample size of preschool-aged children and their parents as participants. Second, we used both direct (cross-classification) and indirect (calculated nutrient intakes) measures to assess the validity of the DAGIS FFQ. Furthermore, we investigated the relative validity of the dietary patterns using both food record data and FFQ rows as inputs. As we did not match the foods in the food records to the FFQ rows for this analysis, we were able to examine the similarity of the derived patterns without artificially overestimating the correlation coefficients. We believe that our approach provided us a more realistic and comprehensive view of the validity of the FFQ.

Our study used food records as the reference method for dietary assessment. However, it is crucial to consider the design of the study critically. FFQs are designed to measure the usual consumption of foods, whereas food records capture only the foods eaten during a few days [1]. Thus, three-day food records may underestimate the consumption of certain foods, such as sugar-containing treats or fish, and FFQs may be able to assess the consumption of these foods more accurately. As dietary patterns are thought to reflect the usual consumption of foods and beverages, FFQs could be thought of as a more accurate assessment method for less frequently used foods. In the present study, the FFQ measured food consumption during the previous week. Thus, the time span did not vary considerably between the two methods (seven days vs. three days), suggesting that the two methods can be meaningfully compared. As our FFQ only measured foods eaten outside of preschool hours, it does not represent the whole diet of the children. Thus, to make the two methods comparable, we excluded foods eaten at preschool from the reference method (three-day food record). However, should the FFQ be used in other studies, it must be noted that the relative validity has only been investigated concerning the foods eaten outside of preschool hours.

As measuring food consumption is not straightforward and every method has its own pitfalls, our measurements were not free of error. It is possible that some foods are most accurately measured using food records, whereas for other foods, the most accurate method might be FFQ. Thus, it must be kept in mind that whenever we are “validating” an FFQ, we are, in fact, just comparing it with another, equally (although differently) biased dietary assessment method, and not the true food consumption, which we unfortunately are unable to assess. Furthermore, the participating families in the DAGIS study were highly educated (in nearly 70% of the families, the highest educational level was bachelor’s degree or higher [24]). As the socio-economic status of the family may be associated with misreporting [33], the results of this study may have to be generalized with caution.

## 5. Conclusions

The FFQ designed for the DAGIS study can rank participants acceptably. Special attention was paid to vegetables and fruits, and sugar-containing foods. The indirect measures of validity (mean intake of selected nutrients in quarters of the consumption) supported the aforementioned conclusion. In addition, three fairly similar dietary patterns were identified using both food-record and FFQ data as inputs, and the percentage of participants classified into the same or adjacent quarters were acceptable. Thus, we conclude that the DAGIS FFQ is a valid measure for vegetable and fruit as well as sugary food consumption. In addition, the DAGIS FFQ can be used to derive dietary patterns that are consistent with those derived using food record data.

## Figures and Tables

**Table 1:** Comparison of food consumption frequencies of foods eaten outside preschool measured with a seven-day food frequency questionnaire (FFQ) and a three-day food record in the Increased Health and Wellbeing in Preschools (DAGIS) study (2015–2016, *n* = 756).

		Consumption Frequency according to FFQ, % of Participants			Comparison of the Two Methods, % of Participants		
Food Item	*n*	Not at All	Less than Daily	Daily	In the Same Quarter	In the same or adjacent quarter	Grossly Misclassified into Opposite Quarters
Vegetables, fruit and berries							
Fresh vegetables	754	2	29	69	35	76	7
Cooked and canned vegetables ^1^	753	25	59	16	35	73	9
Potatoes (in all its forms)	755	3	85	13	30	72	6
Peas, beans, lentils, and soya	754	70	29	1	68	72	13
Fresh fruit	756	2	47	51	38	80	3
Canned and frozen fruit	754	80	20	0.4	78	78	22
Berries	752	30	63	7	41	70	7
Dried fruit and berries	756	58	41	1	61	64	15
Commercial baby foods and smoothies (no added sugar)	755	75	23	1	75	77	14
Berry and fruit soups (with added sugar)	754	64	34	2	66	71	10
Dairy products							
Skimmed milk and sour milk	752	51	6	43	71	96	0.9
1% fat milk, semi-skimmed milk, and sour milk	755	46	8	46	63	94	0.3
Whole milk and sour milk	753	89	7	4	90	90	10
Low-fat cheese (less than 20% fat)	751	60	26	14	59	71	10
High-fat cheese (20% or more fat)	755	33	40	27	50	81	5
Flavored and sweetened milk- and plant-based drinks	755	55	38	7	60	76	12
Natural yoghurt and quark (also plant-based products)	755	63	33	3	67	72	14
Flavored yoghurt and quark (also plant-based products)	754	23	61	16	49	83	3
Puddings	755	67	32	1	69	72	14
Ice cream	754	41	58	1	37	81	10
Fish, meat, and eggs							
Fish dishes and fish products	754	20	79	0.4	29	73	6
Red meat (beef, pork, lamb and mutton, and game)	754	3	91	6	33	65	12
White meat (poultry)	756	13	84	3	35	70	7
Cold cuts	750	35	46	20	43	74	9
Sausages, frankfurters, and luncheon meats	754	23	71	6	31	72	8
Eggs	755	39	60	1	47	71	21
Cereal products							
Brown rice and pasta	755	39	59	2	45	70	12
White rice and pasta	755	30	69	2	33	70	9
Rye bread, crispbread, and thin rye crackers	755	13	55	32	45	75	9
Multigrain bread and wholemeal bread	754	18	59	24	36	74	8
White bread	755	67	28	5	58	63	12
Sugar-sweetened cereals and muesli	754	52	44	3	52	74	10
Berry, fruit, and chocolate porridge (with added sugar)	756	91	9	1	87	87	13
Wholegrain porridge and cereals (no added sugar)	756	31	54	15	46	82	5
Sweet biscuits and cereal bars	755	29	69	2	35	70	9
Sweet pastries ^2^	755	22	77	0.3	27	71	6
Drinks							
Sugar-sweetened juice drinks	752	24	68	8	37	69	8
Fruit juice (no added sugar)	754	54	39	6	58	79	9
Soft drinks (with added sugar)	756	72	28	0.1	72	78	7
Reduced sugar juices and soft drinks	755	87	12	1	85	85	15
Others							
Chocolate	754	35	64	1	33	78	13
Sweets	756	16	83	1	20	78	14
Added sugar, honey, or syrup ^3^	754	59	35	6	62	69	9
Jams, marmalades, and sweetened spreads	755	67	30	3	66	73	9
Plain nuts, almonds, and seeds	754	72	25	3	78	80	11
Flavored nuts, almonds, and seeds (e.g., salted nuts)	755	91	9	0	91	91	9
Crisps and popcorn	756	48	52	0.4	45	60	10

Items in the table are in the same order as in the FFQ (available at https://helda.helsinki.fi/handle/10138/235382). ^1^ As a side dish or as an ingredient in a dish. ^2^ Cakes, cupcakes, sweet rolls, Danish pastries, pies, and other sweet pastries. ^3^ for example, in porridge, tea, berries, yoghurt, or quark.

**Table 2:** Mean daily nutrient intake from foods consumed outside of preschool (three-day food record) according to categories of food group consumption (FFQ) in the DAGIS study (2015–2016, *n* = 756).

	Quarter of Food Group Consumption Frequency according to FFQ				*p*-Value for Trend ^1^
	First	Second	Third	Fourth	
Vitamin C (mg) intake according to vegetable and fruit ^2^ consumption	35	47	54	61	<0.001
Folate intake (µg) according of vegetable and fruit ^2^ consumption	88	91	104	109	<0.001
Calcium intake (mg) according to milk ^3^ consumption	503	610	724	770	<0.001
Sucrose intake (g) according to sugary treats ^4^ consumption	26	29	29	34	<0.001
Sucrose intake (g) according to sugary everyday food ^5^ consumption	25	28	31	35	<0.001
Sucrose intake (g) according to sugary beverage ^6^ consumption	26	28	29	36	<0.001

^1^ Linear regression with food consumption frequency category as a continuous predictor variable; nutrient intakes were square root transformed. ^2^ Sum of the FFQ rows “fresh vegetables”, “cooked and canned vegetables”, “peas, beans, lentils, and soya”, and “fresh fruit’. ^3^ Sum of the FFQ rows “skimmed milk and sour milk”, “1% fat milk, semi-skimmed milk, and sour milk”, and “whole milk and sour milk’. ^4^ Sum of the FFQ rows “ice cream”, “sweet biscuits and cereal bars”, “cakes, cupcakes, sweet rolls, Danish pastries, pies, and other sweet pastries”, “chocolate”, and “sweets”. ^5^ Sum of the FFQ rows “flavored yoghurt and quark”, “puddings”, “sugar-sweetened cereals and muesli”, “berry, fruit and chocolate porridge (with added sugar)”, and “berry and fruit soups (with added sugar)”. ^6^ Sum of the FFQ rows “flavored and sweetened milk- and plant-based drinks”, “sugar-sweetened juice drinks”, and “soft drinks (with added sugar)”.

**Table 3:** Spearman correlation coefficients between the FFQ- and food-record-based dietary pattern scores (strongest positive correlation shown in bold).

	Food-Record-Based Dietary Patterns				
	Health-Conscious	Sandwich	Sweets-and-Treats	Milk, Potatoes and Minced Meat	Pasta, Minced Meat and Fruit
**FFQ-based dietary patterns**					
**Sweets-and-treats**	−0.19 ^1^	−0.18 ^1^	**0.27** ^1^	0.22 ^1^	−0.10 ^1^
**Health-conscious**	**0.33** ^1^	−0.05	−0.30 ^1^	−0.16 ^1^	0.08 ^2^
**Vegetables-and-processed meats**	0.19 ^1^	**0.25** ^1^	0.16 ^1^	0.08 ^2^	0.11 ^1^

^1^*p* ≤ 0.01. ^2^ *p* ≤ 0.05.

**Table 4:** The proportion of participants classified into the same, same or adjacent, or opposite quarters using the FFQ- and food-record-based dietary pattern scores.

	% of Participants Classified into the Same Quarter	% of Participants Classified into the Same or Adjacent Quarters	% of Participants Grossly Misclassified into Opposite Quarters
Sweets-and-treats patterns	34	72	7
Health-conscious patterns	35	73	6
Vegetables-and-processed meats/sandwich patterns	35	73	9

## Supplementary Materials

The following are available online at http://www.mdpi.com/2072-6643/11/1/159/s1: Table S1: Food groups included and excluded in the food-record-based analysis and their mean consumption among 3–6 year-olds in the DAGIS study (2015–2016, *n* = 756); Table S2: Characteristics of the five food-record-based dietary patterns identified among 3–6 year-olds in the DAGIS study (2015–2016).
