# Supplementary material for: Parents’ Reports of Preschoolers’ Diets: Relative Validity of a Food Frequency Questionnaire and Dietary Patterns
Source: Nutrients. 2019 Jan 13;11(1):159. doi: 10.3390/nu11010159 (PMC6356196; doi:10.3390/nu11010159)
Supplement: Supplementary file 1 [file nutrients-11-00159-s001.zip › Supplementary Table S1.docx]

**Supplementary Table S1.** Food groups included and excluded in the food-record-based analysis and their mean consumption among 3–6 year olds in the DAGIS study (2015–2016, n = 756).

| **Food group** | **Consumption in g/day, mean (SD)** | **Included/Excluded in the PCA** |
| --- | --- | --- |
| Vegetables and vegetable dishes, potatoes |  |  |
| Fresh vegetables, vegetable-based salads | 46.5 (40.6) | Included |
| Vegetable soups and sauces, vegetable main course dishes, mushroom dishes | 14.9 (31.2) | Included |
| Vegetable side dishes | 6.1 (12.9) | Included |
| Mayonnaise-based and main course salads | 0.8 (4.4) | Excluded ^1^ |
| Potatoes (boiled, mashed, fried) | 33.1 (35.9) | Included |
| Fruit, berries, fruit and berry dishes |  |  |
| Fresh fruit | 84.1 (72.5) | Included |
| Berries | 8.1 (18.5) | Included |
| Berry and fruit soups | 12.0 (32.9) | Included |
| Berry and fruit pies | 1.5 (6.8) | Excluded ^1^ |
| Canned fruit, fruit salad | 6.7 (22.4) | Included |
| Jams, marmalades | 2.3 (5.8) | Excluded ^1^ |
| Smoothies | 5.6 (23.0) | Included |
| Cereals |  |  |
| Rye bread | 11.8 (14.7) | Included |
| Bread, mixed flour | 15.8 (19.0) | Included |
| Wheat bread, white | 5.2 (10.7) | Included |
| Sugar-sweetened cereals | 5.1 (9.4) | Included |
| Unsweetened cereals | 0.3 (2.7) | Excluded ^1^ |
| Porridge | 60.1 (80.9) | Included |
| Pasta, rice | 23.0 (28.0) | Included |
| Pizza | 5.0 (14.8) | Included |
| Buns, doughnuts, cakes, sweet pastries | 10.4 (16.5) | Included |
| Biscuits | 5.6 (8.9) | Included |
| Savoury pastries, burgers | 10.9 (20.4) | Included |
| Pancakes, crêpes | 4.7 (17.0) | Included |

| Fat spreads, oils, dressings, gravies |  |  |
| --- | --- | --- |
| Margarine, fat blend spread ≥60% | 6.1 (5.8) | Included |
| Margarine, fat blend spread <60% | 0.7 (3.3) | Excluded ^1^ |
| Butter | 0.5 (1.9) | Excluded ^1^ |
| Salad dressings, vegetable oils | 0.3 (1.1) | Excluded ^1^ |
| Gravy and other fat products | 2.0 (7.0) | Excluded ^1^ |
| Fish, meat and egg dishes |  |  |
| Fish, fish fillets, fish casseroles, fish soups, other fish dishes and products | 17.8 (30.7) | Included |
| Cold cuts and sausages, sausage dishes | 13.7 (23.8) | Included |
| Minced meat dishes | 19.6 (26.7) | Included |
| Poultry dishes | 15.3 (22.4) | Included |
| Meat soups, meat casseroles, meat stews | 43.3 (54.9) | Included |
| Steaks, chops | 3.3 (9.2) | Excluded ^1^ |
| Offal dishes | 0.7 (4.0) | Excluded ^1^ |
| Egg dishes | 5.3 (12.3) | Included |
| Milk and dairy, plant-based alternatives |  |  |
| Milk, skimmed | 116.6 (175.0) | Included |
| Milk, 0.1–2% fat | 124.6 (159.9) | Included |
| Milk, >2% fat | 9.8 (52.1) | Included |
| Cream | 0.4 (2.1) | Excluded ^1^ |
| Sour milk, quark | 6.1 (27.2) | Included |
| Flavoured yoghurt | 46.5 (62.0) | Included |
| Natural yoghurt | 9.9 (31.8) | Included |
| Hard cheeses | 6.1 (8.2) | Included |
| Processed and soft cheeses | 3.4 (7.4) | Included |
| Ice cream | 7.5 (13.5) | Included |
| Milk puddings | 15.6 (34.0) | Included |
| Dairy-based sauces | 0.6 (2.6) | Excluded ^1^ |
| Plant-based milks and drinks | 12.8 (72.6) | Included |
| Plant-based creams and desserts | 1.4 (11.3) | Excluded ^1^ |

| Sugar and confectionary |  |  |
| --- | --- | --- |
| Sugar, honey | 0.9 (2.4) | Excluded ^1^ |
| Sweets | 7.5 (13.1) | Included |
| Xylitol-sweetened sweets | 0.4 (0.7) | Excluded ^1^ |
| Chocolate | 3.4 (7.3) | Included |
| Beverages |  |  |
| Fruit juices | 17.9 (45.8) | Included |
| Sugar-sweetened juices | 48.5 (67.0) | Included |
| Artificially sweetened juices | 5.6 (27.6) | Included |
| Sugar-sweetened soft drinks | 9.4 (30.2) | Included |
| Artificially sweetened soft drinks | 3.1 (17.9) | Excluded ^1^ |
| Coffee, tea | 2.6 (18.7) | Excluded ^1^ |
| Water | 79.2 (113.4) | Excluded ^2^ |
| Miscellaneous |  |  |
| Nuts, dried fruits, snacks | 3.9 (7.2) | Included |
| Spices, spice sauces, meal replacement products, other miscellaneous | 3.7 (6.7) | Excluded ^3^ |

^1^ Reason for exclusion: mean consumption less than 10g in three days.

^2^ Reason for exclusion: no nutritional value and inconsistencies in reporting.

^3^ Reason for exclusion: mixed nature of the group (the group contains foods that could not be classified into any of the existing food groups).
