# Supplementary material for: Parents’ Reports of Preschoolers’ Diets: Relative Validity of a Food Frequency Questionnaire and Dietary Patterns
Source: Nutrients. 2019 Jan 13;11(1):159. doi: 10.3390/nu11010159 (PMC6356196; doi:10.3390/nu11010159)
Supplement: Supplementary file 1 [file nutrients-11-00159-s001.zip › Supplementary Table S2.docx]

**Supplementary Table S2.** Characteristics of the five food-record-based dietary patterns identified among 3–6 year olds in the DAGIS study (2015–2016, n = 756).

| **Food group** | **Pattern 1: Health-conscious** | **Pattern 2: Sandwich** | **Pattern 3: Sweets-and-treats** | **Pattern 4: Milk, potatoes and minced meat** | **Pattern 5: Pasta, minced meat and fruit** |
| --- | --- | --- | --- | --- | --- |
| Fruit juices | **0.63** | 0.05 | 0.10 | 0.04 | –0.08 |
| Natural yoghurt | **0.57** | 0.01 | –0.14 | –0.07 | –0.07 |
| Canned fruit, fruit salad | **0.54** | –0.21 | –0.08 | 0.23 | 0.03 |
| Nuts, dried fruits, snacks | **0.26** | –0.10 | 0.04 | 0.01 | 0.10 |
| Margarine, fat blend spread ≥60% | 0.05 | **0.66** | 0.20 | 0.07 | –0.10 |
| Hard cheeses | –0.11 | **0.55** | –0.04 | –0.02 | 0.03 |
| Bread, mixed flour | –0.17 | **0.48** | –0.02 | 0.07 | **0.26** |
| Fresh vegetables, vegetable-based salads | **0.32** | **0.42** | –0.03 | –0.15 | 0.16 |
| Rye bread | **0.25** | **0.40** | –0.06 | –0.14 | **–0.40** |
| Processed and soft cheeses | –0.02 | **0.26** | –0.24 | –0.07 | –0.02 |
| Porridge | 0.20 | –0.21 | **–0.47** | –0.01 | –0.08 |
| Sweets | –0.05 | –0.05 | **0.37** | –0.06 | 0.01 |
| Cold cuts and sausages, sausage dishes | 0.10 | 0.01 | **0.37** | 0.19 | –0.03 |
| Wheat bread, white | 0.05 | 0.04 | **0.35** | –0.07 | 0.20 |
| Sugar-sweetened soft drinks | 0.03 | –0.10 | **0.35** | 0.18 | –0.07 |
| Flavoured yoghurt | –0.04 | 0.14 | **0.34** | 0.22 | –0.08 |
| Berries | 0.21 | 0.04 | **–0.32** | –0.02 | 0.20 |
| Sugar-sweetened juices | 0.03 | –0.06 | **0.31** | –0.18 | 0.00 |
| Fish, fish fillets, fish casseroles, fish soups, other fish dishes and products | 0.13 | 0.13 | **–0.28** | –0.15 | 0.17 |
| Chocolate | –0.12 | –0.10 | **0.27** | –0.19 | –0.02 |
| Berry and fruit soups | –0.01 | 0.07 | **–0.27** | 0.06 | –0.18 |
| Sour milk, quark | 0.14 | 0.11 | **–0.27** | 0.09 | 0.05 |
| Milk, 0.1–2% fat | –0.16 | 0.12 | –0.10 | **0.56** | –0.13 |
| Milk, skimmed | **0.40** | 0.10 | 0.17 | **–0.51** | 0.12 |
| Potatoes (boiled, mashed, fried) | **0.31** | 0.12 | 0.07 | **0.46** | –0.02 |
| Minced meat dishes | –0.06 | 0.07 | 0.03 | **0.41** | **0.41** |
| Vegetable soups and sauces, vegetable main course dishes, mushroom dishes | –0.02 | 0.06 | –0.08 | **–0.40** | –0.02 |
| Pasta, rice | –0.07 | –0.05 | 0.00 | 0.08 | **0.58** |
| Meat soups, meat casseroles, meat stews | 0.00 | 0.02 | –0.05 | –0.03 | **–0.56** |
| Fresh fruit | 0.08 | 0.18 | –0.13 | –0.23 | **0.30** |
| Vegetable side dishes | 0.13 | 0.08 | –0.06 | 0.06 | **0.26** |
| Poultry dishes | 0.04 | –0.15 | 0.10 | –0.07 | **0.26** |
|  |  |  |  |  |  |
| Eigenvalue | 2.08 | 1.77 | 1.73 | 1.64 | 1.62 |
| % of total variance explained | 4.6 | 3.9 | 3.8 | 3.6 | 3.6 |

Altogether, 13 food groups included in the analysis (smoothies; sugar-sweetened cereals; pizza; buns, doughnuts, cakes, sweet pastries; biscuits; savory pastries, burgers; pancakes, crêpes; egg dishes; milk, >2% fat; ice cream; milk puddings; plant-based milk and drinks; artificially sweetened juices) did not load with an absolute value of 0.25 or more to any of the five patterns identified and are not shown in the table. Loadings with an absolute value of 0.25 or more are shown in bold.

.
